# Expanding the Spectrum of *CSF3R*-Mutated Myeloid Neoplasm Beyond Chronic Neutrophilic Leukemia and Atypical Chronic Myeloid Leukemia: A Comprehensive Analysis of 13 Cases

**DOI:** 10.3390/jcm14155174

**Published:** 2025-07-22

**Authors:** Neha Seth, Judith Brody, Peihong Hsu, Jonathan Kolitz, Pratik Q. Deb, Xinmin Zhang

**Affiliations:** 1 Department of Pathology and Laboratory Medicine, Donald and Barbara Zucker School of Medicine at Hofstra/Northwell, Greenvale, NY 11548, USA; nseth1@northwell.edu (N.S.);; 2 Department of Medicine, Northwell Cancer Institute, Donald and Barbara Zucker School of Medicine at Hofstra/Northwell, Manhasset, NY 11030, USA

**Keywords:** *CSF3R*, myeloid neoplasm, myelodysplastic/myeloproliferative neoplasm, acute leukemia, myelodysplastic neoplasm

## Abstract

**Background:** Genetic alterations in *CSF3R*, typically associated with chronic neutrophilic leukemia (CNL) and atypical chronic myeloid leukemia (aCML), rarely occur in other myeloid neoplasms. **Methods:** This study characterized the clinical, morphologic, cytogenetic, and molecular features of 13 patients with non-CNL non-aCML myeloid neoplasms with *CSF3R* alterations. Patients (median age, 77 years) were categorized into groups with a myelodysplastic/myeloproliferative neoplasm (MDS/MPN) (n = 5), acute leukemia (n = 4), and other myeloid neoplasms (n = 4) based on the WHO 2022 and ICC criteria. **Results:** The *CSF3R* p.*Thr*618*Ile* mutation was most frequent (11/13), with additional pathogenic variants including p.*Gln*743*Ter* and frameshift mutations affecting the cytoplasmic tail. Variant allele frequencies (VAFs) ranged from 2% to 49%, with the highest median VAF in the MDS/MPN group. Co-mutations varied by subtype; MDS/MPN, NOS, and CMML cases frequently harbored mutations in epigenetic regulators (*ASXL1*, *TET2*) and splicing factors (*SF3B1*, *SRSF2*, *ZRSR2*), while acute leukemia cases showed alterations in *JAK3*, *STAT3*, and *NRAS*. Survival analysis revealed distinct patterns across the three diagnostic groups, with MDS/MPN having the poorest prognosis. **Conclusion:** This study expands the recognized spectrum of *CSF3R*-related myeloid neoplasms and highlights the clinical and molecular heterogeneity associated with these mutations, emphasizing the need for comprehensive molecular profiling and the potential for targeted therapies.

## 1. Introduction

The colony-stimulating factor 3 receptor (CSF3R) is a key member of the hematopoietic receptor super-family. The protein CSF3R, encoded by the eponymous gene (*CSF3R*), plays a crucial role in the proliferation, differentiation, and survival of granulocytes [1,2]. Upon binding its ligand granulocyte colony stimulating factor (G-CSF), *CSF3R* triggers various downstream pathways, including the JAK-STAT pathway, leading to its biological effects [3]. Due to this key biological function in hematopoiesis, genetic alterations in *CSF3R* are strongly linked to granulocytic dysfunction. The T618I mutation in *CSF3R*, in particular, is the most frequent genetic alteration that leads to constitutive receptor activation, resulting in aberrant JAK-STAT, SRC family kinase, and RAS-MAPK signaling, driving granulocytic proliferation and cytokine-independent cell survival [4,5].

Somatic activating *CSF3R* mutations are highly associated with chronic neutrophilic leukemia (CNL) and present with neutrophilia, hypercellular bone marrow with granulocytic proliferation, and the absence of dysplasia or increased blasts. This rare myeloproliferative neoplasm (MPN) usually shows an indolent clinical course, although the outcome may vary [6,7]. CNL may acquire additional mutations and progress to blast crisis with or without the acquisition of dysplasia; however, the specific alteration in *CSF3R* persists in the progressing disease, often with increased allele frequency, suggesting a linear molecular progression of this disease [8,9]. In addition to CNL, *CSF3R* alterations are frequently seen in atypical chronic myeloid leukemia (aCML), also known as myelodysplastic/myeloproliferative neoplasm with neutrophilia (MDS/MPN-N), and severe congenital neutropenia (SCN) [10,11]. Alterations in *CSF3R* are also infrequently associated with other myeloid neoplasms, such as chronic myelomonocytic leukemia (CMML), myelodysplastic neoplasms (MDS), or acute myeloid leukemia (AML) [12].

Although *CSF3R* genetic alterations may belong to three different classes, the point mutation T618I, affecting the transmembrane proximal domain, is the most common pathogenic alteration in myeloid neoplasms. This mutation often acts as the driver mutation in CNL, persisting throughout the disease progression [8]. Importantly, CNL patients with the T618I mutation show a significantly worse prognosis compared to those with other *CSF3R* mutations [13]. While this mutation may not independently drive leukemogenesis, it may play a synergistic role in disease pathogenesis. Its ability to constitutively activate the JAK-STAT pathway has made it a rational therapeutic target, with JAK inhibitors such as ruxolitinib having been successfully used to treat neoplasms driven by this alteration [11,14,15]. All these factors make the identification of myeloid neoplasms with *CSF3R* alteration clinically relevant.

In this study, we examined the clinical, hematological, histomorphological cytogenetic, and molecular characteristics of 13 cases of non-CNL, non-aCML myeloid, and non-SCN myeloid neoplasms. We further documented the mainstay therapy that patients received and their outcome. By investigating these rare cases, we have explored whether there are any underlying unifying characteristics in these neoplasms.

## 2. Materials and Methods

### 2.1. Patient Selection and Data Collection

We searched our institutional database for all pathology diagnostic reports for “*CSF3R*”. All retrieved cases were manually reviewed independently by two board-certified hematopathologists. Patients were included if they harbored a *CSF3R* mutation confirmed by next-generation sequencing (NGS), had a clinical and hematopathology diagnosis that did not meet the criteria for CNL, aCML, or SCN, and had comprehensive molecular, cytogenetic, and morphologic data available. Of all such cases, only newly diagnosed myeloid neoplasms were included in this study. Demographic and clinical data, including age, sex, clinical presentation, and laboratory findings, were extracted from the institutional electronic medical record system manually.

### 2.2. Hematologic and Morphologic Analysis

Complete blood counts, peripheral blood smears, and bone marrow aspirates/core biopsies were reviewed. Dysplasia across erythroid, myeloid, and megakaryocytic lineages was documented.

### 2.3. Cytogenetic and FISH Analysis

Conventional karyotyping and fluorescence in situ hybridization (FISH) were performed for all cases as part of standard diagnostic evaluation.

### 2.4. Molecular Studies

Targeted NGS was performed through an OnkoSight myeloid panel (BioReference Health^TM^) comprising up to 50 genes, which included *ABL1*, *ANKRD26*, *ASXL1*, *ATRX*, *BCOR*, *BCORL1*, *BRAF*, *CALR*, *CBL*, *CCND2*, *CDKN2A*, *CEBPA*, *CSF3R*, *CUX1*, *DDX41*, *DNMT3A*, *ETNK1*, *ETV6*, *EZH2*, *FBXW7*, *FLT3*, *GATA2*, *HRAS*, *IDH1*, *IDH2*, *JAK2*, *KDM6A*, *KIT*, *KMT2A*, *KRAS*, *MAP2K1*, *MPL*, *MYD88*, *NF1*, *NPM1*, *NRAS*, *PDGFRA*, *PHF6*, *PTEN*, *PTPN11*, *RUNX1*, *SETBP1*, *SF3B1*, *SRSF2*, *STAG2*, *TET2*, *TP53*, *U2AF1*, *WT1*, and *ZRSR2*. The OnkoSight Myeloid panel used has a validated analytical sensitivity of 1–2% VAF for SNVs and small indels, with a minimum read depth > 500×. Only variants that passed internal quality control filters were included in the final analysis.

### 2.5. Statistical Analysis

Descriptive analysis was employed to summarize clinical and laboratory data. All statistical analyses including Kaplan–Meier survival analysis and data visualizations were performed using Python 3.14^TM^. Tables, and all visualizations with histograms, box plots, bar charts, heatmaps, and lollipop plots were generated with libraries such as Matplotlib, Seaborn, and Pandas.

### 2.6. Ethical Considerations

This study was conducted in accordance with institutional review board (IRB) policies regarding the use of patient data in research. Patient identifiers were anonymized to ensure confidentiality.

## 3. Results

### 3.1. Clinical Findings

We examined the molecular profiles of 1400 patients with myeloid neoplasms diagnosed at our institute over a seven-year period. We identified thirteen (0.9%) with *CSF3R*-mutated myeloid neoplasms, which were categorized into three diagnostic groups. The MDS/MPN group includes three cases of MDS/MPN-unclassified (MDS/MPN-U) and two cases of CMML; the acute leukemia group includes two cases of AML, one case of mixed phenotype acute leukemia (MPAL)-M/T, and one case of myeloid sarcoma (MS). The third group includes three cases of de novo MDS and one case of MPN with progression to MDS. The median age for the whole cohort was 77 years (range, 22 to 89 years), with a male predominance (nine males, four females). Patients in the MDS/MPN group were predominantly elderly (median age, 84 years; range, 78 to 89 years). The acute leukemia cases spanned a wide age range (22 to 69 years; median, 56 years). Patients in the other myeloid neoplasm group had a median age of 68 years (range, 57 to 79 years). An elevated lactate dehydrogenase (LDH) level (≥242 U/L) was present in 11 out of 13 patients, and splenomegaly was seen in 2 patients. Clinical history was significant for previous malignancy (four cases), autoimmune disease (three cases), and hepatitis (one case) (Table 1).

### 3.2. Peripheral Blood Findings

For the whole cohort, the median white blood cell (WBC) count was 14.6 × 10^9^/L (range, 1.4 × 10^9^/L to 58.5 × 10^9^/L), with both leukopenia and leukocytosis observed. Hemoglobin (Hb) levels ranged from 7.3 g/dL to 12.0 g/dL, (median, 9.4 g/dL), with severe anemia (Hb < 8.0 g/dL) noted in four patients. Platelet counts ranged from 27 × 10^9^/L to 216 × 10^9^/L (median, 95 × 10^9^/L). Patients in the MDS/MPN group presented with leukocytosis and varying degrees of anemia, and thrombocytopenia (3/5 cases). Peripheral blood smears revealed neutrophilia (>80% of leukocytes), with immature granulocytes comprising <10% of the WBC count in all cases. Absolute monocytosis (>1 × 10^9^/L and ≥10% of cells) were noted only in the CMML patients. There was no increase in basophils or blasts in any case. Peripheral smears in the acute leukemia group exhibited varying degrees of leukocytosis and cytopenia, with circulating blasts ranging from 1% to 77%. Peripheral smears in the other myeloid neoplasm group revealed normocytic anemia, anisopoikilocytosis, thrombocytopenia (3/4 cases), and dysgranulopoiesis.

### 3.3. Histopathological Findings in Bone Marrow

Bone marrow biopsies in the MDS/MPN group consistently revealed marked hypercellularity (70 to 100% cellularity) with myeloid predominance in three cases and erythroid predominance or a normal M/E ratio in each of the remaining two cases. Dysplasia is noted in all cases. All but one case (patient 4) had less than 5% blasts. Ring sideroblasts exceeding 15% were observed in two cases (Case 1 and Case 4). Moderate to marked reticulin fibrosis (grade 2–3) was seen in two patients (Case 2 and Case 3).

Bone marrow aspirates and biopsies in the acute leukemia patients revealed hypercellularity (70% to 85% cellularity) with extensive blast infiltration and decreased normal hematopoiesis in three cases. The bone marrow in the myeloid sarcoma case showed myeloid hyperplasia, left-shifted granulopoiesis, and dyserythropoiesis and the extramedullary site involved (the lymph node) showed the effacement of the architecture by a diffuse proliferation of immature myeloid cells, consistent with MS.

The bone marrow findings in the other myeloid neoplasm group ranged from normocellular to markedly hypercellular marrow (30 to 100% cellularity), with myeloid predominance in 3 out of 4 patients, and dysplasia in at least one lineage in all patients. Increased blasts (5 to 8%) were noted in the bone marrow in all patients.

**Figure 1:**
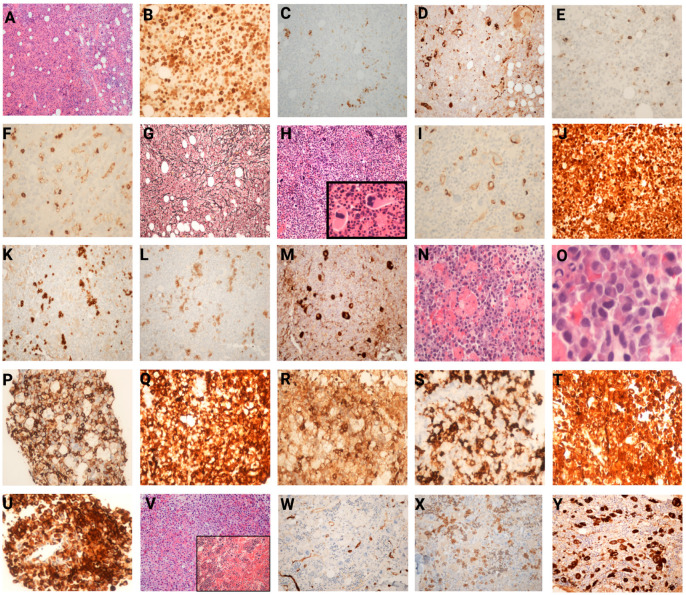
Histomorphological and immunohistochemical features of myelodysplastic/myeloproliferative neoplasms. (**A**–**G**) Photomicrographs showing features of the bone marrow of an MDS/MPN-U patient with *CSF3R* alteration. (**A**) Photomicrograph showing bone marrow with marked hypercellularity, myeloid predominance, and decreased erythroid and megakaryocytic populations. (**B**) Myeloperoxidase and (**C**) CD71 confirm myeloid predominance. (**D**) Factor VIII IHC showing megakaryocytes with atypia. (**E**) CD34 IHC demonstrating <5% CD34-positive blasts. (**F**) CD14 IHC highlighting an increased monocytic population. (**G**) Reticulin stain showing grade 2-3 myelofibrosis. (**H**–**M**) Photomicrographs showing features of the bone marrow of a CMML patient with *CSF3R* alteration. (**H**) Photomicrograph of a bone marrow biopsy showing markedly hypercellular marrow with significant granulocytic hyperplasia and a prominent left shift in myeloid maturation (H&E, 40×, inset 100×). (**I**) CD34 IHC demonstrating less than 5% blasts and megakaryocytes with aberrant CD34 expression. (**J**) Myeloperoxidase, (**K**) CD71, and (**L**) E-cadherin confirm myeloid predominance and left shift in the erythroid compartment. (**M**) Factor VIII highlights dysplastic megakaryocytes. (**N**–**U**) Photomicrographs showing features of myeloid sarcoma with *CSF3R* alteration. Photomicrograph showing an atypical proliferation of pleomorphic hematopoietic cells with necrosis and apoptosis with (**N**) H&E, 40× and (**O**) H&E, 400×. Neoplastic cells are positive for (**P**) CD45, (**Q**) CD4, (**R**) CD33, (**S**) MPO, (**T**) CD163, and (**U**) Muramidase (IHC, 40×). (**V**–**Y**) Photomicrographs showing features of bone marrow in patient with essential thrombocythemia gaining a secondary *CSF3R* alteration. (**V**) Photomicrograph showing a markedly hypercellular bone marrow (H&E, 20×) with left-shifted myeloid maturation and inset showing increased atypical megakaryocytic forms (H&E, 400×). (**W**) CD34 immunohistochemical stain highlighting the sinusoids and several megakaryocytic forms indicative of dyspoiesis (IHC, 100×). (**X**) E-cadherin immunohistochemical stain showing the prominence of pronormoblasts (IHC, 100×). (**Y**) Factor VIII marking the increased megakaryocytes with atypia and occasional clustering (IHC, 100×, 400×).

The histomorphological and immunophenotypic findings from the bone marrow biopsies of representative cases are depicted in Figure 1.

### 3.4. Cytogenetic and FISH Analysis

Among the 13 cases, normal karyotypes were observed in 8 cases. Abnormalities included monosomy 7 (Case 2), deletion 7q (Case 4), a complex karyotype in Case 9 (44-45,X,-Y,der(4)t(4;6)(p12;p11.2)der(4)(q31),-8,-9,+mar1,+mar2[6]/46,XY [cp14]), and a derivative chromosome involving chromosomes 13 and 14 in Case 12. Case 13 demonstrated a 45,-X,-Y karyotype. FISH studies identified monosomy 7 in Case 2 and deletion 7q in Case 4, confirming karyotype findings. The amplification of the *RUNX1* gene was detected in Case 6 (three copies), and a RUNX1T1/RUNX1 rearrangement was identified in Case 9. FISH was normal in seven cases; results were unavailable in two cases. One patient with MDS/MPN-U had an isolated deletion of the Y chromosome, likely an age-related finding.

### 3.5. Molecular Findings

The mutational landscape across the 13 patients with *CSF3R*-mutated myeloid neoplasms was highly heterogeneous. All patients harbored *CSF3R* alterations, with variant allele frequencies (VAFs) ranging from 2% to 49%, indicating varying degrees of clonal involvement.

**Figure 2:**
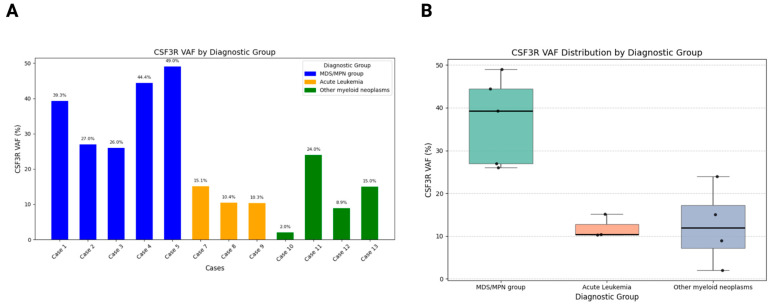
(**A**) This bar chart illustrates the VAF of *CSF3R* mutations across individual cases, categorized by diagnostic groups. Blue bars represent cases classified as being in the MDS/MPN group, which demonstrate intermediate-to-high *CSF3R* VAF values (median, 39%; range, 26–49%). Orange bars represent cases diagnosed as acute leukemia (median, 10%; range, 10–15%), which tend to exhibit lower *CSF3R* VAF values compared to the MDS/MPN group. Green bars represent cases diagnosed for other myeloid neoplasms, which show varying levels of *CSF3R* VAF (median, 12%; range, 2–24%). (**B**) Box plot showing the *CSF3R* variant allele frequency (VAF%) across three diagnostic groups: MDS/MPN group, acute leukemia, and other myeloid neoplasms.

Grouped analysis revealed that patients with MDS/MPN-U and CMML demonstrated the highest *CSF3R* VAFs (median, 39%; range, 26–49%), consistent with a predominant clonal driver role. In contrast, cases with acute leukemia exhibited the lowest VAFs (median, 10%; range, 10–15%), suggesting a possible subclonal or secondary role of *CSF3R* in leukemogenesis. Other myeloid neoplasms displayed intermediate VAFs (median, 12%; range, 2–24%), indicating a smaller contribution of *CSF3R* to disease pathogenesis. These differences are visualized in Figure 2A,B, which display VAF distributions across diagnostic groups using bars and box plots, respectively.

**Figure 3:**
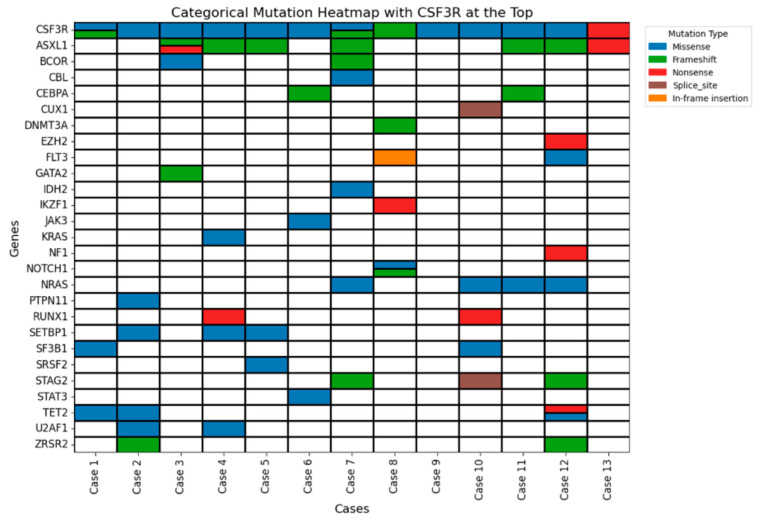
The heatmap highlights the mutation patterns and recurrently altered genes seen across 13 cases of myeloid neoplasms. Colored boxes represent mutation types: blue for missense, green for frameshift, red for nonsense, and brown for splice-site mutations, and orange for in-frame insertions. White boxes indicate no mutation.

Co-mutations varied by disease subtype and are depicted in a categorical mutation heatmap (Figure 3). The most common co-mutations were *ASXL1* and mutations in the RAS signaling pathway (*NRAS*, *KRAS*, and *PTPN11*). MDS/MPN-U and CMML patients frequently carried mutations in epigenetic regulators (*ASXL1*, *TET2*) and RNA splicing genes (*SF3B1*, *SRSF2*, *ZRSR2*). A *SETBP1* mutation was identified in one patient with CMML. In the acute leukemia group, *CSF3R* mutations co-occurred with alterations in signaling pathway genes, including *JAK3*, *STAT3*, and *NRAS*. For instance, Case 6 (AML with biallelic *CEBPA* mutations) also harbored the *STAT3* mutation. Other myeloid neoplasms, including MDS, showed low-frequency *CSF3R* mutations (≤10%), suggesting a smaller subclonal role. Interestingly, *KRAS* mutations were observed in two of three MDS cases, possibly indicating an underlying MDS/MPN-like biology.

The most frequent *CSF3R* mutation was the canonical p.Thr618Ile missense mutation in exon 14, identified in 11 cases. Three additional pathogenic mutations were detected: a nonsense mutation (p.Gln743Ter) in two cases and two frameshift mutations (p.Lys785SerfsTer26 and p.S810Qfs) affecting the cytoplasmic tail of the protein in three cases. Case 1 carried both p.Thr618Ile and p.S810Qfs mutations. Only two patients had an isolated *CSF3R* mutation; one of these was of AML with a *RUNX1*::*RUNX1T1* translocation, suggesting that *CSF3R* was not the driver mutation in these two cases.

**Figure 4:**
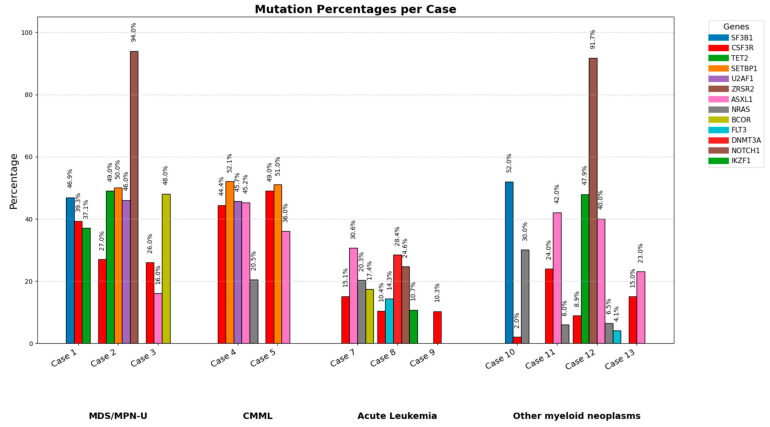
**Genetic mutation frequencies in *CSF3R*-driven myeloid neoplasms**. This bar chart illustrates the percentage frequencies of key representative genetic mutations identified in various cases with *CSF3R*-driven myeloid neoplasms showcasing the heterogeneity of these disorders. Case 6, which harbors *CSF3R*, *STAT3*, *CEBPA*, and *JAK3* mutations, is excluded from this graph, as percentage data for these mutations are not available.

The mutation landscape was visualized using a stacked bar chart showing mutation percentages per case (Figure 4). Across the cohort, the most frequently mutated genes included *ASXL1, NRAS, SETBP1*, and *TET2*. Cases 4 and 5 demonstrated the highest mutation burden, with *CSF3R* mutations comprising 44.4% and 49% of total mutations, respectively. In contrast, Cases 6–9 (acute leukemia) exhibited lower overall mutation percentages, with no single gene exceeding 30% VAF. The stacked bars also reveal patterns of co-mutations in individual cases, such as the presence of multiple gene mutations in Cases 1, 4, and 5 (MDS/MPN-U and CMML), compared to a more restricted mutational profile in some acute leukemia cases. This diversity in mutation percentages highlights the complex clonal architecture of *CSF3R*-mutated myeloid neoplasms and emphasizes the importance of considering the broader mutational context when interpreting the role of *CSF3R* in disease pathogenesis and therapeutic decision-making.

### 3.6. Clinical Outcomes

**Figure 5:**
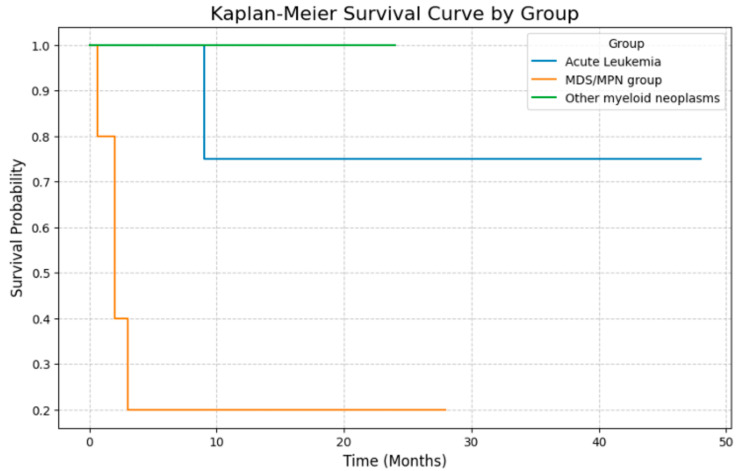
The Kaplan–Meier survival curve illustrates the survival probabilities of three diagnostic groups: MDS/MPN group (orange), acute leukemia (blue), and other myeloid neoplasms (green), over time in months. The MDS/MPN group shows a steep decline in survival, reflecting the poor prognosis in disorders like MDS/MPN-U and CMML. The acute leukemia group exhibits a more gradual decrease, indicating variable outcomes in aggressive diseases like AML, MPAL, and myeloid sarcoma. The other myeloid neoplasms demonstrate relatively stable survival, consistent with their indolent nature. Vertical ticks on the curves mark censoring, representing patients still alive or lost to follow-up.

Survival data were available for all patients across the three diagnostic categories: MDS/MPN, acute leukemia, and other myeloid neoplasms. Kaplan–Meier analysis demonstrated distinct survival patterns among the groups. The median survival for the entire cohort was approximately 12 months. The MDS/MPN group exhibited the steepest decline in survival, with most patients succumbing to the disease early in its course. The acute leukemia group had a more gradual decline, reflecting variable clinical trajectories. In contrast, the other myeloid neoplasms group, comprising mainly MDS-IB1, had a more stable survival curve, consistent with slower disease progression. Survival curves for each group are shown in Figure 5.

## 4. Discussion

Our study highlights the significant presence of the *CSF3R* mutation across a diverse range of myeloid neoplasms, expanding their known spectrum beyond the traditional associations with CNL, aCML, and severe congenital neutropenia (SCN) [16]. In our cohort of thirteen patients, we identified *CSF3R* alterations across a variety of myeloid neoplasms, including MDS/MPN-U, AML, MPN with disease progression, MPAL, CMML, and MDS-IB1, suggesting a broader role for *CSF3R* in pathogenesis of myeloid neoplasms and clonal evolution. Given the rarity of *CSF3R*-mutated myeloid neoplasms outside of CNL and aCML, our cohort represents one of the largest institutional case series described to date. However, the small sample size inherently limits the statistical power of subgroup comparisons and survival analyses. Therefore, our findings should be interpreted as exploratory and hypothesis-generating, requiring validation in larger, multi-institutional cohorts.

**Figure 6:**
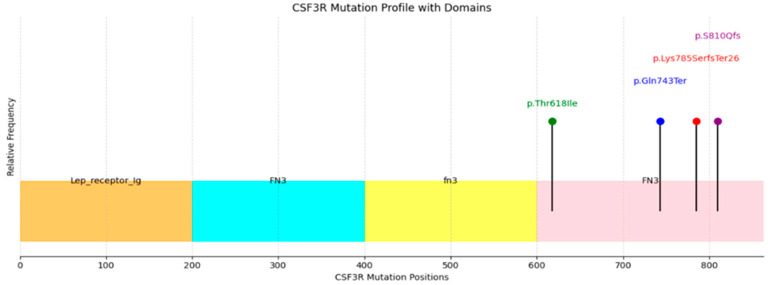
Lollipop plot conveying *CSF3R* mutation profile with functional domains and hotspot mutations.

Functionally, these mutations affect distinct receptor domains. The membrane-proximal missense mutation p.*Thr*618*Ile* impairs self O-glycosylation, resulting in ligand-independent receptor dimerization and continuous signaling in the JAK-STAT signaling pathway [17]. In contrast, truncation mutations (p.*Gln*743*Ter*, p.*Lys*785*Ser*fsTer26) and frameshift mutations (p.S810Qfs) cluster within the cytoplasmic tail and disrupt negative regulatory motifs responsible for receptor internalization and degradation, thereby prolonging receptor activity and preferentially activating the SRC/TNK2 signaling pathway and enhanced cell proliferation [11,18,19]. The lollipop plot shown in Figure 6 illustrates their distribution across different functional domains.

While the *CSF3R* T618I mutation remains a hallmark of CNL (reported in up to 83% of cases) and is also present in aCML [17], our findings reinstate that this mutation is not restricted to these entities. This functional divergence emphasizes its distinct roles in myeloid pathogenesis and aligns with prior studies showing that *CSF3R* truncation mutations are frequently associated with severe disease phenotypes and resistance to therapy [5,20]. While their location in the cytoplasmic tail suggests the disruption of key regulatory domains, these effects remain inferential. Future studies utilizing predictive modeling or cellular assays will be necessary to clarify their true biological and clinical impact.

We also found frequent co-occurrences of *CSF3R* mutations with alterations involving other genes including, but not limited to, *ASXL1*, *NRAS*, *KRAS*, and *SETBP1* in the MDS/MPN-U and CMML patients. These co-mutations likely contribute to the phenotypic heterogeneity and clinical variability observed in these *CSF3R*-driven neoplasms [21]. Among these, *ASXL1* mutations were the most frequent co-mutations in our cohort, consistent with their known association with poor prognosis and disease progression in myeloid malignancies [13,22]. This suggests that *ASXL1* co-mutations might exacerbate the effects of *CSF3R* mutations by promoting epigenetic dysregulation. The *ASXL1* mutation, in combination with proliferative mutations such as *ETNK1* and *SETBP1*, bring about the MDS/MPN phenotype in aCML. It is likely that the combination of *ASXL1* and *CSF3R* creates a similar phenotype with the same mechanism. *NRAS* and *KRAS* mutations, observed in a few cases, highlight the role of aberrant RAS-MAPK signaling in driving clonal proliferation and leukemic transformation in these neoplasms [23].

In their study of *CSF3R* alteration in CNL and CMML, Ouyang and colleagues found that *SRSF2* and *SETBP1* were associated with a worse prognosis, whereas *CSF3R* alteration did not affect outcomes [24]. Other studies have suggested a role of *SETBP1* alteration as secondary drivers of leukemic transformation and resistance to therapy [25]. In our dataset, both *SETBP1* (Case 2, 3, and 5) and *SRSF2* (Case 5) were noted predominantly in the MDS/MPN subcategory and were associated with poorer outcomes.

Amongst acute leukemia patients in our cohort, one patient had *RUNX1::RUNX1T1* translocation, while another had a biallelic *CEBPA* mutation, both of which are known to co-occur with *CSF3R* alterations [26,27]. This pattern implies that in these settings, *CSF3R* mutations may contribute to disease progression. It is also important to note that none of the patients in our cohort received JAK inhibitor therapy, primarily due to advanced age, comorbidities, or limited clinical access at the time of diagnosis. As a result, while the therapeutic potential of *CSF3R*-targeted therapies such as ruxolitinib is discussed, actual treatment response data could not be assessed in this study.

Clinically, the identification of *CSF3R* mutations has profound therapeutic implications, emphasizing the need for routine molecular profiling in myeloid malignancies to identify *CSF3R* mutations and associated co-mutations. This is particularly relevant in patients without clearly defined category (MDS/MPN-U), where the presence of p.Thr618Ile or truncation mutations could provide a therapeutic target. The association of *ASXL1*, *SRSF2*, and *SETBP1* mutations with poor prognosis, as evidenced by other studies, underscores the need for comprehensive molecular profiling [28]. As previously discussed, the constitutive activation of signaling pathways like JAK-STAT and SRC/TNK2 in *CSF3R*-altered neoplasms presents opportunities for targeted interventions with documented efficacy using JAK inhibitors such as ruxolitinib in these neoplasms [11,29]. Additionally, inhibitors targeting downstream pathways, such as SRC family kinases, could complement JAK inhibitors in cases where alternative signaling predominates. These therapeutic approaches could potentially be combined with other emerging therapeutic approaches such as MEK inhibitors to target RAS pathway or DNA methyltransferase inhibitors in *ASXL1*-mutated neoplasms.

From a practical standpoint, while *CSF3R* mutations are rare in myeloid neoplasms outside of CNL and aCML (~0.9% in our series), they remain clinically actionable. In resource-limited settings where broad NGS testing may not be available, focused hotspot testing for canonical mutations like p.Thr618Ile in patients presenting with neutrophilic leukocytosis or MDS/MPN-like features could offer a cost-effective approach. Developing such context-specific testing algorithms may optimize molecular diagnostics and facilitate access to targeted therapies, even in settings with limited resources.

## 5. Conclusions

Our study provides valuable insights into the genetic landscape of *CSF3R*-mutated myeloid neoplasm and adds to the growing body of evidence that *CSF3R* mutations, especially T618I, are a critical component of the molecular landscape beyond CNL, aCML, and SCN. We identified these mutations across a spectrum of myeloid disorders, including MDS/MPN, AML, and other myeloid disorders, reinforcing their relevance across diagnostic categories.

While our findings suggest that *CSF3R* mutations may assist in disease stratification and represent potential therapeutic targets, these conclusions are limited by the small sample size and lack of statistically significant outcome associations in this study. Therefore, we propose that *CSF3R* mutations be considered as potential markers of interest rather than definitive stratifiers at this stage. Further multicenter studies with larger cohorts and integrated clinical, genomic, and treatment data will be essential in validating their prognostic utility and defining their role in personalized therapeutic strategies.

## Figures and Tables

**Table 1:** Comprehensive clinicopathologic, cytogenetic, and outcome profile of the 13-patient myeloid neoplasm cohort.

	Case 1	Case 2	Case 3	Case 4	Case 5	Case 6	Case 7	Case 8	Case 9	Case 10	Case 11	Case 12	Case 13
Age	89	81	87	84	78	22	69	55	56	57	77	63	79
Gender	F	M	F	M	M	M	F	M	M	F	M	M	M
WBC	58.5	14.6	44	16.2	51.9	25.5	4.2	6.1	1.4	10.4	2.6	25.3	2.5
Hb	10.6	7.3	7.4	9.2	11.7	11.7	12	11	7.6	9.4	10.6	7.7	11.2
MCV	99.7	100.4	103.9	85.5	105.5	94.6	81	86.2	92.6	90.9	84.3	98.7	96.3
Plt	216	27	141	32	158	47	184	95	44	117	280	77	65
Mono(%)	3	6	NA	10	12.1	0	9	1	4	5.2	NA	2.9	9
Mono#	1.2	0.9	2.3	1.8	6.3	0	0.4	0	0.1	0.54	NA	0.7	0.22
PB blasts (%)	0	0	0	0	0	85	0	63	14	0	NA	0	0
LDH	385	220	531	418	888	406	337	314	666	416	1269	1176	ND
Cellularity	90	85–90	95	70–85	100	70–85	Limited	95–100	70	95	90	95–100	30
M/E	10:1	MP	MP	1:1	2:1	0.8:1	3:1	MP	5.2:1	6.5:1	1.6:1	10.8:1	MP
Erythroid	Dys+	Dys+	Dec	Dys+	Dec	Dys+	Dec	Dec	Dec	Dys+	Dys+	Inc	Mild dec
Myeloid	NA	NA	Inc	Dys+	Inc	Minimal	Dec	Dec	Dec	Dys+	Dys+	Rare	Dys+
Megakaryocytes	NA	Dys+	Dec	Dys+	Inc, Dys+	No	Rare	Dec	Present	Dys+	Dys+	Dys+	Dec
BM blasts (%)	<3	<3	<3	7	1	66	4	83	56	8	8	8	5
Fibrosis	0	Gr 2-3	Gr 2-3	0	0	0	0	0	0	0	0	Gr 1-2	0
Karyotype	46, XX	45,XY,-7	46, XX	46,XY del(7)(q22)	46, XX	46, XY	46, XX	46,XY	Complex	46,XX	46,XY	46,XY,der(13;14)9q10;q10)	45, -X,-Y
FISH	Normal	Monosomy 7	NA	Del(7q)	Normal	RUNX1 (3 copies)	Normal	Normal	RUNX1T1/RUNX1	Normal	Normal	NA	Normal
Diagnosis	MDS/MPN-U	MDS/MPN-U	MDS/MPN-U	CMML	CMML	AML	MS	MPAL	AML	MDS-EB1	MDS-EB1	ET/MPN transformed to AML	MDS-EB1
Treatment	Hy 500 mg OD	Azax1 cycle	NA	Supportive care only	Hy 500 mg OD	7+3+GO, Consolidation HiDAC + GO × 4	7+3; HiDAC + Aza × 2; Ven + Dec × 2	7+3	7+3	No Rx	Ven + Dec × 4; GO+ LD Ara-C	Ven + Dec; ASCT	No Rx
Prognosis	Died, 33 months	Died, 2 months	2 months	Died, 19 days	Alive, 28 months	CR, 4 years	Relapse, 14 months	Died, 9 months	Relapse, 19 months	Alive, 1 year	Transform into AML, alive, 2 years	CR, 1 year	Alive, 5 months
Clinical	None	Hepatitis, cirrhosis	Breast Ca, colon Ca	Prostate Ca	B-ALL, remission	None	RA	DVT	None	OA knee	MG	None	Prostate Ca
Splenomegaly	No	Yes	Yes	No	No	No	No	No	No	No	No	No	No

Abbreviations: MDS/MPN-U, Myelodysplastic syndrome/Myeloproliferative neoplasm—Unclassifiable; HA, hypomethylating agent; Hy, hydroxyurea; Dys, dysplasia; Inc, increased; Dec, decreased; MS, myeloid sarcoma; RA, rheumatoid arthritis; MG, Myasthenia gravis; OA, osteoarthritis; DVT, deep vein thrombosis; MP, myeloid predominant; Rx, treatment; OD, once daily; Aza, Azacytidine; Dec, Decitabine; HiDAC, high-dose cytarabine; 7+3, cytarabine + Daunorubicin; Ven, Venetoclax; GO, Gemtuzumab Ozogamicin; LD, low-dose; Ara-C, Cytarabine; ASCT, allogenic stem cell transplant; NA, not available.

## Data Availability

All data and information concerning this study will be made available from the corresponding authors upon reasonable request.
